# Visual Schema Displacement Therapy versus Eye Movement Desensitization and Reprocessing therapy versus waitlist in the treatment of post-traumatic stress disorder: results of a randomized clinical trial

**DOI:** 10.3389/fpsyt.2024.1377108

**Published:** 2024-03-26

**Authors:** Suzy J. M. A. Matthijssen, Thomas C. Brouwers, Ad de Jongh

**Affiliations:** ^1^ Altrecht Academic Anxiety Centre, Altrecht Geestelijke Gezondheidszorg (GGz), Utrecht, Netherlands; ^2^ Psychotrauma (PSYTREC), Bilthoven, Netherlands; ^3^ Academic Centre for Dentistry Amsterdam (ACTA), University of Amsterdam and Vrije Universiteit Amsterdam, Amsterdam, Netherlands; ^4^ School of Health Sciences, University of Salford, Manchester, United Kingdom; ^5^ Institute of Health and Society, University of Worcester, Worcester, United Kingdom; ^6^ School of Psychology, Queen’s University, Belfast, United Kingdom; ^7^ Research Department, Psychotrauma (PSYTREC), Bilthoven, Netherlands

**Keywords:** post-traumatic stress disorder (PTSD), Visual Schema Displacement Therapy (VSDT), Eye Movement Desensitization and Reprocessing (EMDR) therapy, trauma treatment, randomized controlled trial (RCT)

## Abstract

**Introduction:**

Visual Schema Displacement Therapy (VSDT) is a novel approach showing promise in mitigating distressing memories, akin to Eye Movement Desensitization and Reprocessing (EMDR).

**Objectives:**

This study aimed to determine the safety, feasibility, and effectiveness of VSDT in individuals with post-traumatic stress disorder (PTSD), comparing it to EMDR therapy and a waitlist control condition (WLCC). It was hypothesized that the application of VSDT would be safe and PTSD symptoms significantly be reduced from both baseline to post-treatment and from baseline to follow-up in the VSDT and EMDR therapy conditions. Furthermore, we expected both treatments to be significantly more effective than the waitlist control. Moreover, we hypothesized that VSDT and EMDR therapy would be associated with significant improvements in symptoms of depression and general psychopathology.

**Method:**

Forty-six adults with PTSD were randomly assigned to VSDT, EMDR therapy, or WLCC, receiving six 90-minute sessions. Assessments included the Clinician Administered PTSD Scale for the Diagnostic Statistical Manual (DSM)-5 (CAPS-5), PTSD Checklist for DSM-5 (PCL-5), Beck Depression Inventory-II (BDI-II) and Brief Symptom Inventory (BSI) before, during, and 3 months post-treatment.

**Results:**

Bayesian analysis found no differences between VSDT and EMDR in PTSD symptom reduction but both outperformed WLCC. EMDR was superior to the WLCC in reducing symptoms of depression and general psychopathology. At 3-month follow-up, 58.3% of the participants in the VSDT condition no longer met the PTSD diagnostic criteria (41.2% EMDR therapy and 15.4% WLCC) with no difference between the two therapy conditions. Self-reported PTSD symptom reduction was significant in VSDT (d = 1.38) and EMDR (d = 1.40) but modest in WLCC (d = 0.39). Dropout rate was 19.3%, with no adverse events.

**Conclusion:**

This study supports VSDT’s efficacy in treating PTSD, offering a valuable therapeutic option comparable to EMDR, with significant reductions in PTSD symptoms and no difference with EMDR or the control condition for depressive symptoms and general psychopathology, and no reported adverse events.

## Highlights

- Visual Schema Displacement Therapy (VSDT) seems effective in reducing symptoms of post-traumatic stress disorder (PTSD).
- Visual Schema Displacement Therapy (VSDT) is capable of resolving post-traumatic stress disorder (PTSD).
- Eye Movement Desensitization and Reprocessing (EMDR) and Visual Schema Displacement Therapy (VSDT) elicit equal effects in the treatment of post-traumatic stress disorder (PTSD).

## Introduction

Post-traumatic stress disorder (PTSD) is a debilitating mental health condition which one can develop after exposure to one or more traumatic events (1). Fortunately, there are a wide array of evidence-based therapies for the treatment of PTSD (2, 3). As remission rates are still not 100% (4) there is an ongoing search for enhancement of existing trauma treatments, unraveling the working mechanisms of existing therapies, but also a quest for new and promising therapies. One of these new and potentially promising therapies is Visual Schema Displacement Therapy (VSDT; 5). Although the application of VSDT differs from that of many other therapies, the core of the treatment method is aimed at reducing the disturbance of aversive, or otherwise emotional memories so that reprocessing can take place and connections can be created with more adaptive information, aligned with the idea of the adaptive information processing model (6).

A paper written about the first studies that investigated the efficacy of VSDT was published in 2019 (5) and described how VSDT was tested using two analogue studies. The first study used a within-group design and compared an Eye Movement Desensitization and Reprocessing (EMDR) therapy condition to either VSDT or a control condition. The second experiment employed a between-group design with the same conditions, allowing follow-up measurement to be taken into account (for more information on EMDR therapy and the proposed working mechanism, see 7). In both studies VSDT and EMDR therapy proved superior to the control condition in reducing emotional disturbance, and VSDT was found to be superior to EMDR therapy. Furthermore, both VSDT and EMDR therapy outperformed the control condition in terms of reducing vividness. Given that EMDR therapy is recommended as one of the evidence-based treatments for PTSD (e.g., 8, 9), the results warranted further research into potential working mechanisms and randomized clinical trials carried out in patients with PTSD. The former was conducted using a study that tested several potential working mechanisms (i.e., counterconditioning, the presence of a surprise element inducing arousal and the importance of line of sight) within a non-clinical sample (7). Again, VSDT proved effective in decreasing emotionality and outperformed both EMDR therapy and the control condition, but at four-week follow-up VSDT and EMDR therapy yielded similar effects. In vividness both VSDT and EMDR therapy outperformed the control condition. None of the assumed working mechanisms appeared to play a vital role, or at least not on its own (7).

Since previous studies have focused on determining effectiveness within a non-clinical sample and had emotionality and vividness as outcome measures (5, 7), it remains unclear whether the effects can also be replicated using a clinical sample, and whether the effects on emotionality and vividness also translate into effects on PTSD symptomatology and comorbid symptoms. Therefore, the present study used a randomized controlled trial design to test safety, feasibility, and effectiveness of VSDT in people with PTSD, in comparison with EMDR therapy as an active control condition and a waitlist condition to control for natural recovery. We hypothesized that VSDT would be safe and feasible to carry out as a clinical psychotherapy (i.e., no adverse events would occur). Further, we expected that PTSD symptoms would be significantly reduced from baseline to post-treatment and from baseline to 3-month follow-up, and that both would be significantly more effective than the waitlist control condition. In comparing VSDT and EMDR head-to-head, we expected no statistical differences due to insufficient statistical power to detect small effect sizes. We hypothesized that both VSDT and EMDR therapy would be associated with an improvement in comorbid psychopathology at baseline, post-treatment and follow-up.

## Methods

### Design

The study was based on a three (Condition: VSDT, EMDR therapy, waitlist control) by three (time: pretreatment, post-treatment, and follow-up) mixed design. Following inclusion, participants were randomly allocated to one of the three conditions based on a randomly generated list of numbers and inclusion order with an allocation ratio of 1:1:1. The within-subjects factor Time was defined by the total scores of the Clinician Administered PTSD Scale for the Diagnostic Statistical Manual (DSM) -5 (CAPS-5) measured at three time points: during screening (pretreatment), at one month posttreatment and at 3-month follow-up. The research assistants who conducted the post-treatment and follow-up measurements were blinded to the conditions. Furthermore, the self-report questionnaires PTSD Checklist for DSM-5, Beck Depression Inventory-II and Brief Symptom Inventory (respectively PCL-5, BDI-II and BSI) were completed weekly during study participation for descriptive purposes, and as a secondary outcome measure at the same time points as the CAPS-5. Details regarding the treatment sessions (e.g., Subjective Unit of Disturbance (SUD) scores and duration) were also logged during sessions.

### Participants

Participants were recruited at the Altrecht Academic Anxiety Centre from Altrecht GGz, a large regional mental healthcare facility in the Netherlands which offers specialized mental health care. Participants were informed by their therapist about the possibility of participating, either following the intake procedure as an alternative to waiting for regular treatment, or as a modular treatment option while receiving other, non-PTSD related treatment. The inclusion criteria were as follows: meeting the DSM-5 criteria for PTSD according to the Clinician Administered PTSD Scale for DSM-5 (CAPS-5; 10, 11), being aged 18 years or older, not being suspected of mental disability, and having sufficient command of the Dutch language to follow Dutch treatment protocols. The exclusion criteria consisted of the actual presence of a high level of suicidal risk according to the Mini-International Neuropsychiatric Interview (M.I.N.I.) section C (12, 13), a change in psychopharmacological medication three months prior to or during participation, the use of benzodiazepines, currently receiving another PTSD treatment, the use of any recreational drugs a month prior to or during participation, alcohol use exceeding two standard drinks on a daily basis during participation, and alcohol use on the day before, on and after treatment sessions. We did not exclude patients in this study based on the type of trauma or comorbidity (e.g., personality disorders and psychosis).

**Figure 1:**
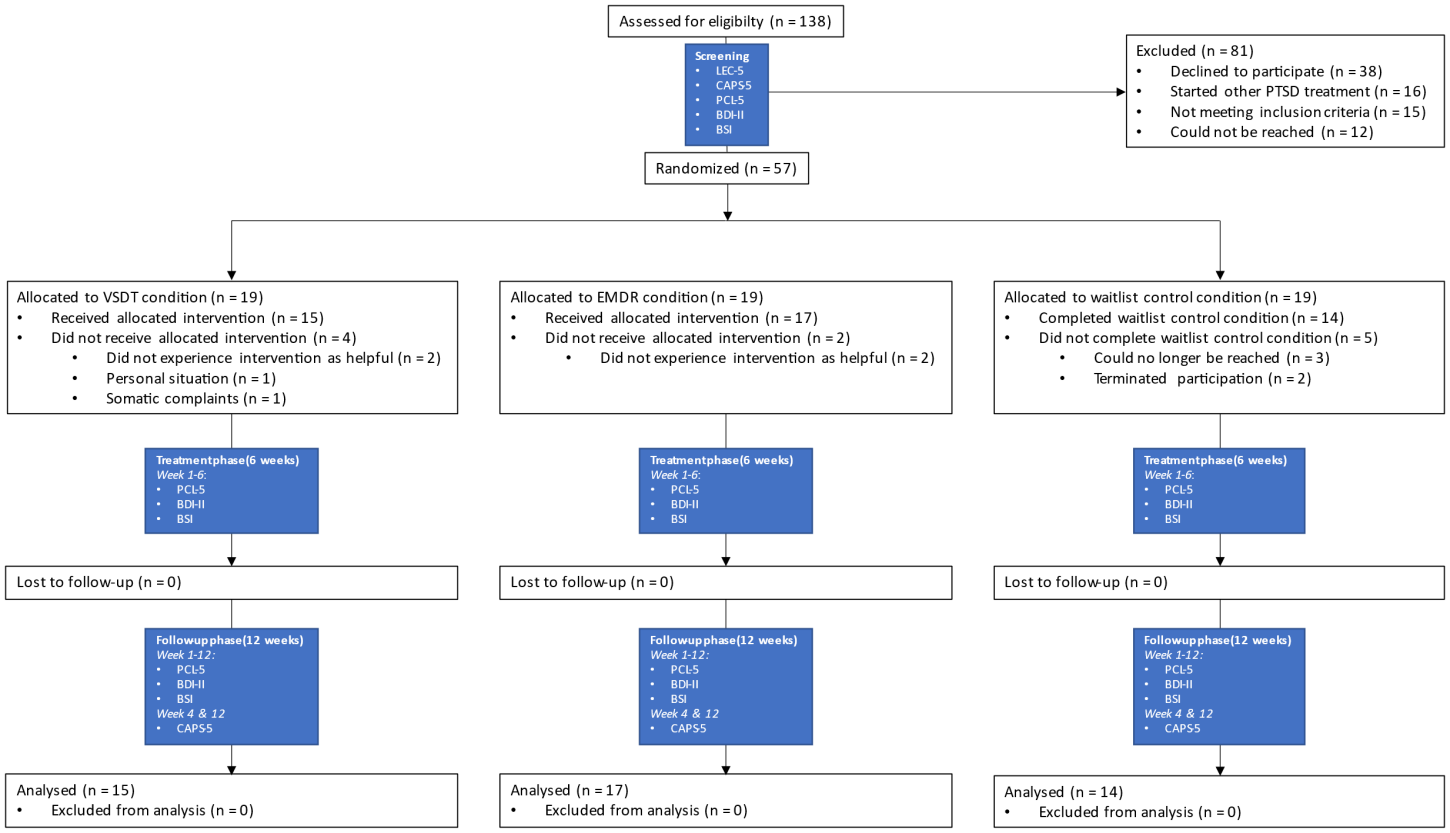
CONSORT Flow Diagram and measurement overview. EMDR, Eye Movement Desensitization and Reprocessing; VSDT, Visual Schema Displacement Therapy; PTSD, post-traumatic stress disorder; LEC-5, Life Event Checklist for DSM-5; CAPS-5, Clinician-Administered PTSD Scale for DSM-5; PCL-5, PTSD Checklist for DSM-5; BDI-II, Beck Depression Inventory-II; BSI, Brief Symptom Inventory.

Although Bayesian statistics do not require an *a priori* power analysis, they were computed and preregistered in the OSF (https://osf.io/xvwe9), recommending a total sample size of N = 51. Recruitment continued for nearly three years until 57 participants were included, thereby accounting for an approximately 10% dropout rate. The participants did not receive any monetary compensation for their participation, or any other benefits, except for the possibility of receiving PTSD treatment more quickly than regular treatment routes. A CONSORT flow diagram is shown in **Figure 1**.

### Procedures

All study procedures were reviewed and approved by the Medical Research Ethics Committee (Protocol ID:19-171/D) and (pre)registered in the Dutch National Trial Register (NTR) and Open Science Framework (OSF; https://osf.io/xvwe9). No changes were made to the study methods after the trial commencement. The study consisted of three phases: screening (one appointment), treatment (six weekly sessions), and follow-up (12 weeks). To ensure allocation concealment, all screening, randomization and planning procedures were performed by the study coordinator, one of the authors (TB), who did not provide any treatment sessions or follow-up measurements. All study appointments were conducted at the Altrecht Academic Anxiety Centre.

Upon showing initial interest, potential participants were contacted via telephone by the study coordinator to provide verbal explanation of the study details. Subsequently, the participants received an information letter containing detailed study information. Another telephone appointment was scheduled, to provide potential participants with sufficient time to fully read the information letter and consider participation. When all participants’ questions had been answered during the following appointment and they still wanted to participate, a screening appointment was planned. Written informed consent for participation in the trial was obtained during this appointment. Furthermore, the patients completed a screening questionnaire assessing the inclusion and exclusion criteria and demographic variables, followed by the assessment of the Life Events Checklist DSM 5 (LEC-5) and CAPS-5, and upon definitive inclusion, the assessment of the weekly study questionnaires (PCL-5, BDI-II, and BSI; see Measurements). Allocation to both treatment conditions and waiting lists occurred immediately after the screening appointment.

In the case of a treatment condition, seven appointments (i.e., one preparation appointment and six subsequent treatment sessions) were planned at the earliest availability, usually 2–4 weeks after the screening appointment. The treatment phase was initiated during the first treatment session. In the case of the waitlist control condition, the treatment phase began a week after the screening appointment. The follow-up phase began immediately after completion of the treatment phase. The therapist’s involvement ceased at the start of the follow-up phase, and responsibility for their mental health care returned to the referring therapist. Participants completed three weekly self-report questionnaires (PCL-5, BDI-II, BSI) during all treatment and follow-up weeks and were invited for post-treatment and follow-up measurement with the CAPS-5 in weeks 4 and 12 after treatment was completed, respectively (see **Figure 1**). Self-report questionnaires were administered digitally using the web-based software LimeSurvey (14). The first was completed after inclusion during the screening appointment, during which questions were asked by the research coordinator, whereas the other 18 were completed independently by the participants weekly during the treatment phase of the study (six weeks) and the follow-up phase (12 weeks) using a weblink on their own device (see **Figure 1**). After completion of the final weekly measurement and the follow-up CAPS-5 measurement, participants were debriefed and clinically relevant outcomes from the interviews were shared with their therapist to evaluate treatment progress.

## Materials

### Clinical interview (CAPS-5)

The main outcome variables for this study were severity of PTSD symptoms and fulfilling the diagnostic criteria of PTSD as indexed by the Dutch version of the Clinician Administered PTSD Scale for DSM-5 (CAPS-5; 10, 11). This structured clinical interview consists of 20 items on the frequency and intensity of PTSD symptoms, scored on a 5-point Likert scale from 0 (*absent*) to 4 (*extreme/incapacitating*), with a maximum score of 80. A score of 2 or higher indicates a clinically relevant symptom, and can be taken into account when determining a PTSD diagnosis following the DSM-5 algorithm (1, 10, 11). It was conducted during the screening appointment, both as a screening and as a pretreatment measurement, by the study coordinator who was officially trained in its use. The interview was repeated at posttreatment and follow-up measurements 4 and 12 weeks after completion of the treatment phase (see **Figure 1**). Follow-up measurements were conducted by several diagnostically trained psychologists with extensive experience with the CAPS-5. The participants were blinded to condition allocation.

### Self-report questionnaires

PCL-5. The PTSD Checklist for DSM-5 (PCL-5) is a validated and widely used self-report measure of PTSD symptoms. It includes 20 items corresponding to the symptoms in the criteria for PTSD according to the DSM-5, which are scored on a 5-point Likert scale from 0 (*not at all*) to 4 (*extremely*), with a total score ranging from 0 to 80 (15, 16). Psychometric evaluations demonstrate high internal consistency and good validity (17). This research in a US veteran sample suggests a score of 31–33 may indicate clinically relevant PTSD symptoms.

BDI-II. The Beck Depression Inventory-II (BDI-II) is a 21-question self-report inventory questionnaire used to assess the severity of depressive symptoms (18, 19). Each symptom is rated on a scale ranging from 0 (*absent*) to 3 (*severe*). Total scores of 0–13 indicate no to minimal depressive symptoms, 14–19 mild depressive symptoms, 20–28 moderate depressive symptoms and 29–63 severe depressive symptoms.

BSI. The Brief Symptom Inventory (BSI) is a multidimensional questionnaire measuring general psychopathology (20, 21). Fifty-three psychiatric symptoms are measured on a 5-point Likert scale ranging from 0 (*not at all present*) to 4 (*present a lot*). The subscales include items on somatic complaints, cognitive problems, interpersonal sensitivity, depressive mood, anxiety, hostility, phobic anxiety, paranoid thoughts and psychoticism. A total score is calculated by averaging all symptoms. The Dutch norm scores for the BSI are an average total score of 1.22 (*SD* = 0.73) for psychiatric patients and 0.38 (*SD* = 0.34) for the general population.

### Treatment

The treatment phase consisted of seven weekly 90-minute sessions. The first appointment was used to establish a treatment plan consisting of the traumatic history and the selection of six most disturbing traumatic memories as targets for subsequent sessions. Memories were treated in order of disturbance, from most to least disturbing based on the SUD (22). If a target reached a SUD of 0 with more than 15 minutes session time left, the next target on the treatment plan was treated until the session time was over. The exact session time (i.e., the number of minutes the treatment protocol was executed) was recorded manually by the therapists using a stopwatch. If all targets were treated before completion of the six sessions and no other relevant traumatic memories could be selected, the treatment phase would end^1^.

Five licensed mental healthcare psychologists working at Altrecht GGz were trained in the treatment protocols by two of the authors (AdJ for EMDR therapy and SM for VSDT) of whom AdJ is a licensed trainer for the EMDR Europe Association. Further supervision was provided in regular sessions with therapists and trainers during which video recordings of the treatment sessions were viewed and evaluated. In addition, a summarized report from each session was emailed to the respective trainers who provided written feedback. Adherence to the treatment protocols was ensured by protocolized fidelity checks, performed by two master’s students who were trained for this purpose and blinded to the treatment outcome. They evaluated a total of 31 videotapes (16%) using treatment-specific checklists. The videotapes were randomly selected and stratified on treatment arm (VSDT = 15; EMDR therapy = 16) and therapist, with the restriction that each stratum did not include duplications of session and participant numbers. Treatment fidelity was high for both conditions (VSDT = 97.64%; EMDR therapy = 88.21%).

### VSDT

VSDT was executed using the Standard VSDT protocol, developed by two of the authors (SM and AdJ), in cooperation with the originators of VSDT, Nik and Eva Speakman (23). All five therapists were trained in the VSDT protocol by SM. After general instructions, VSDT starts with the assessment of the “laughter point,” during which patients are asked to select a mental representation of a person or a memory of an event that made them laugh and were requested to select a keyword for this memory/person. The therapist then holds a watch with the dial facing the patient and draws a circle with a diameter of approximately 1.5 meter in a clockwise motion from the patients’ point of view. The patient is instructed to indicate where the strongest urge to laugh was felt in the circle. This point (the laughter point) is indicated with the keyword and the patient indicates the urge to laugh at the laughter point on a scale of 0 (“no urge to laugh”) to 10 (“maximum urge to laugh”). Next, the emotional disturbing memory is assessed following the same procedure, where the patient indicates the “trauma point” on the location in the circle where the most disturbance is felt, which is subsequently rated from 0 to 10 (analogue to the SUD scale; 22). After successful assessment of both the laughter and trauma points the therapist continues to the displacement phase. During this phase, the patient is instructed to keep his or her eyes focused on the watch and the therapist subsequently moves the watch quickly from the trauma point to the laughter point while saying out loud “Whoosh!”, thereby aiming to startle the patient. Next, the patient is instructed to blink quickly while being primed with the keyword of the laughter point. The displacement procedure is performed in a sequence of three repetitions, followed by patients tightly squeezing their eyes twice and exhaling two deep sighs. The patient then rerates how much the perceived emotionality of the disturbing memory has declined compared to the previous rating, after which the displacement procedure is repeated (in case of SUD > 0) or completed (in case of SUD = 0).

### EMDR therapy

EMDR therapy was executed according to the Dutch translation of the EMDR Standard protocol, based on the standardized eight-phase protocol (for an in-depth description of EMDR therapy, see: 24, 25). The patients were not taught stabilization (i.e., emotion regulation) techniques prior to treatment (for rationale, see 26). All five therapists completed training in EMDR therapy accredited by EMDR Europe, and three therapists were licensed EMDR Europe Practitioners.

### Data analysis

Procedures for data analyses were preregistered in the Open Science Framework (OSF; https://osf.io/xvwe9). Bayesian statistics were used for all analyses, computed using the statistical software JASP (v0.16.4; 27). Bayesian analyses use the Bayes Factor (BF) to signify relative support for one hypothesis or model compared to a null hypothesis or multiple other hypotheses or models. A BF > 1 indicates support for the tested hypothesis, and a BF of 0–1 indicates support for the null hypothesis or alternative models. This is also the main advantage of Bayesian statistics; its possibility to state evidence *for* both the null and alternative hypotheses, thereby providing more extensive information than a single *p*-value stating evidence *against* the null hypothesis, thereby strengthening the credibility of an analysis (28). Furthermore, the absence of a specific threshold value (i.e., *p* = .05) prevents bias in statistical or publication-related decision making.

Bayesian repeated measures analyses of variance (ANOVA) were used to analyze group differences over time (pretreatment, posttreatment, follow-up) for the main (CAPS-5) and secondary outcome measures (PCL-5, BDI-II, BSI). Effects were further specified by *post hoc* analyses of slope differences using ANOVAs with condition as independent variable and difference scores of the outcome measures between the different time points as dependent variable. Loss of diagnosis was analyzed using McNemar proportional analysis. For comparison of treatment-specific continuous variables, independent-samples t-tests (ISTT) were used. Bayesian ANOVAs and Bayesian multinomial (2x2 contingency table) tests were used to analyze distribution of all relevant variables (continuous and categorical respectively) at pretreatment to control for successful randomization. In reporting the ANOVAs, BF_M_ quantifies the support for a single model versus the other tested models; these models include the main effects for Time and Condition, and the interaction effect between those two. When testing a single hypothesis, BF_10_ expresses support for the tested hypothesis versus the null hypothesis, and vice versa for BF_01_. To guide the reader in interpreting the BFs; one can consider a BF of 1–3 as minor support, 3–10 as moderate support and >10 as major support. The default priors for the analyses of variance were based on the work of Rouder et al. (29). Analyses were automatically corrected in JASP for multiplicity by fixing to 0.5 the prior probability that the null hypothesis holds across all comparisons, similar to the Bonferroni correction in NHST (30, 31). Because Bayesian statistics have become more common in psychological publications we decided to deviate from the preregistration and only analyze the data using Bayesian analyses to prevent double reporting of the results. Multinomial contingency table analyses of categorical variables with more than two variables and McNemar’s test are not computable with Bayesian statistics in JASP, therefore, frequentist analyses were used in these cases.

## Results

### Descriptive statistics and randomization check

Data from 46 participants were analyzed. The flow of participants is shown in **Figure 1**. Participants had a mean age of 33.28 years (*SD* = 10.94); 95.7% identified themselves as female, 4.3% as male. The average total pretreatment CAPS-5 score was 41.76 (*SD* = 9.98) and the self-reported PCL-5 pretreatment mean score was 53.57 (*SD* = 10.09; indicating severe PTSD symptoms). They further had a BDI-II mean score of 35.59 (*SD* = 10.16; indicating severe depressive symptoms) and a BSI mean score of 2.10 (*SD* = 0.66; indicating severe psychiatric symptoms). Randomization was successful, as there were no differences in these pretreatment scores between the conditions for CAPS-5 (BF_01_ = 3.90), PCL-5 (BF_01_ = 1.90), BDI-II (BF_01_ = 2.96), and BSI (BF_01_ = 2.16). Age and sex were also successfully randomized between the conditions (BF_01_ = 4.25, *p* >.05, respectively). The results showed no statistical difference between the number of participants treated by the therapists (BF_01_ = 1.06), and the treatment conditions were successfully randomized within each therapist (*p* >.05).

### Safety and treatment drop out

No adverse events occurred over the course of the study. The observed dropout rates in our study exceeded our initial expectations. Specifically, the dropout rate reached 19% (whereas our *a priori* estimate was 10%. Nevertheless, it is noteworthy that the randomization process was effective. It is crucial to acknowledge that the consequence of a reduced sample size, resulting from the elevated dropout rate, will inevitably manifest as diminished Bayes Factors in our statistical analyses. Bayes Factors are indicative of the strength of evidence in favor of one hypothesis over another in the context of Bayesian statistics.

### Main analyses

#### Clinical interview (CAPS-5)

**Figure 2:**
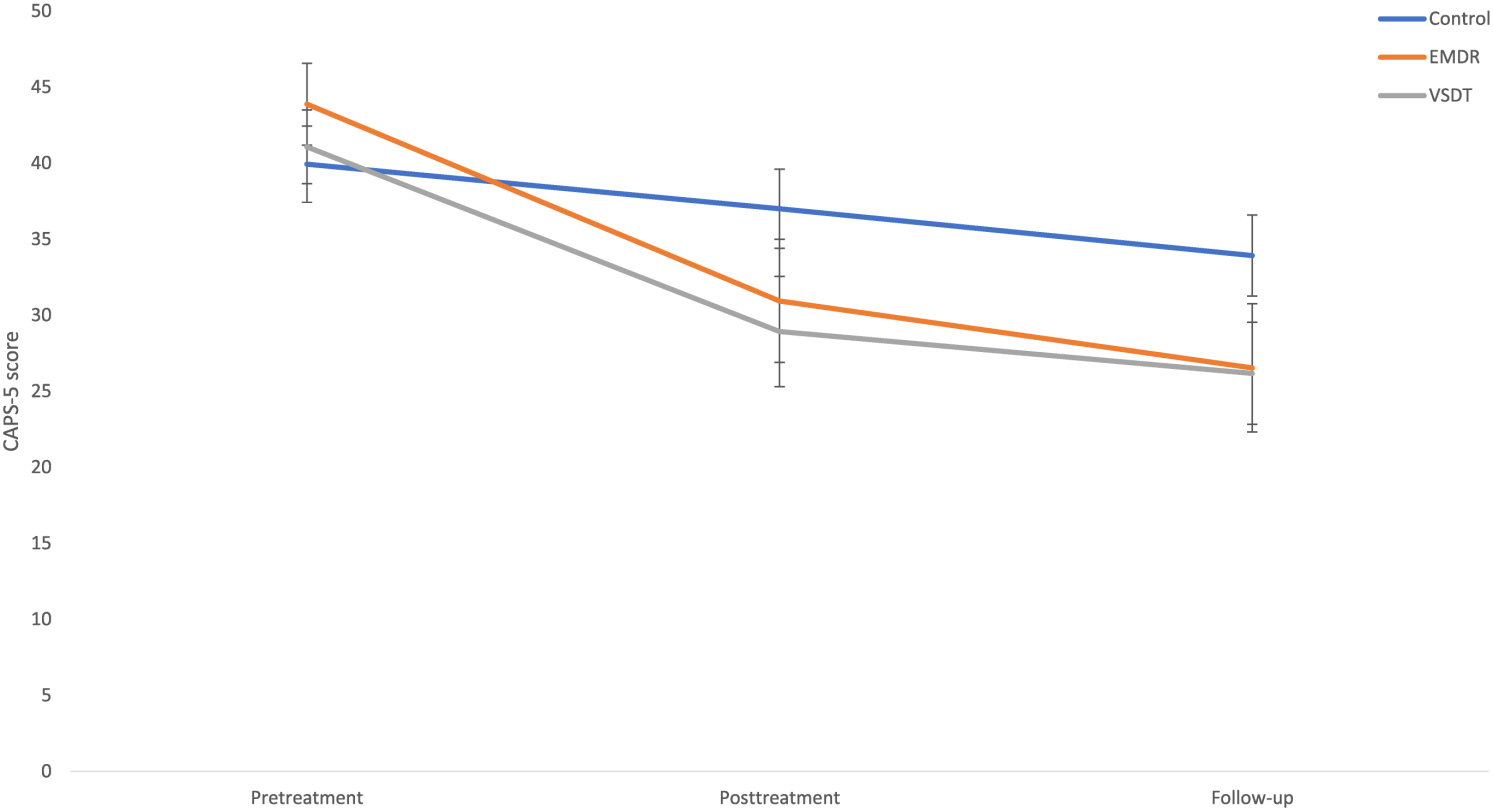
Mean (SE) CAPS-5 scores specified per condition and timepoint. EMDR, Eye Movement Desensitization and Reprocessing; VSDT, Visual Schema Displacement Therapy; CAPS-5, Clinician Administered PTSD Scale for DSM-5.

PTSD symptom severity. A Bayesian repeated measures ANOVA comparing CAPS-5 scores over time (pretreatment, posttreatment, and follow-up) between conditions (VSDT, EMDR therapy, Control) shows the most support for the model including only a main effect for Time (B_M_ = 5.31), when compared to the model including both the main effect for Time and Condition (B_M_ = 1.37), the model including both main effects and the interaction effect (B_M_ = 0.84), and the model including only the main effect for Condition (B_M_ = 9.02 x 10^−7^). The *post hoc* ANOVA comparing difference scores between groups from pretreatment to posttreatment shows support for the null-hypothesis (BF_01_ = 1.73). For pretreatment to follow-up, roughly equal support for both hypotheses is found (BF_10_ = 1.05), and for posttreatment to follow-up the null-hypothesis is supported (BF_01_ = 3.54). The only relevant slope differences that can be detected, although minimally supported, are a larger decrease in the VSDT condition from pretreatment to posttreatment (BF_10_ = 1.57) compared to the control condition, and a larger decrease in both the VSDT (BF_10_ = 1.43) and EMDR therapy (BF_10_ = 1.86) conditions from pretreatment to follow-up when compared to the control condition. For a (graphic) overview of these results, see **Table 1** and **Figure 2**.

**Table 1:** Means and Standard Deviations for primary and secondary outcome measures specified per condition and compared across timepoints.

	EMDR									VSDT									Control								
	Pre	Post	FU	Pre-Post		Pre-FU		Post-FU		Pre	Post	FU	Pre-Post		Pre-FU		Post-FU		Pre	Post	FU	Pre-Post		Pre-FU		Post-FU	
	*M (SD)*	*M (SD)*	*M (SD)*	*BF*	*d*	*BF*	*d*	*BF*	*d*	M (SD)	M (SD)	M (SD)	*BF*	*d*	*BF*	*d*	*BF*	*d*	*M (SD)*	M (SD)	M (SD)	*BF*	*d*	*BF*	*d*	*BF*	*d*
**CAPS-5**	43,88 (11.10)	30.94 (16.19)	26.53 (17.39)	6.74	0.77	36.79	0.97	1.29	0.51	41.07 (9.38)	28.93 (14.04)	26.17 (11.63)	22.45	0.99	19.12	1.13	0.29	0.03	39.93 (9.39)	37.00 (9.38)	33.92 (9.62)	1.31	0.56	0.91	0.48	0.73	0.44
**PCL-5**	57.06 (10.21)	34.43 (19.82)	30.94 (21.80)	166.46	1.38	519.58	1.40	0.30	0.12	51.40 (9.51)	35.11 (16.80)	30.92 (14.18)	23.24	1.50	104.27	1.38	0.66	0.47	51.64 (10.07)	50.77 (17.05)	45.15 (15.99)	0.30	0.12	0.62	0.39	0.77	0.46
**BDI-II**	37.53 (10.24)	26.07 (14.06)	22.06 (14.17)	37.85	1.12	332.36	1.33	0.38	0.24	32.53 (9.90)	30.00 (15.32)	26.92 (11.30)	0.47	0.33	0.56	0.36	0.42	0.26	36.50 (10.32)	34.92 (13.97)	30.54 (16.87)	0.37	0.22	1.06	0.52	0.53	0.35
**BSI**	2.30 (0.70)	1.44 (0.95)	1.29 (0.96)	22.92	1.04	59.06	1.08	0.28	0.07	1.90 (0.74)	1.60 (0.99)	1.41 (0.74)	1.00	0.59	0.98	0.50	0.52	0.37	2.08 (0.48)	2.09 (0.86)	1.78 (0.92)	0.28	0.02	0.82	0.46	0.62	0.40

FU, Follow-up measurement 12 weeks posttreatment; EMDR, Eye Movement Desensitization and Reprocessing; VSDT, Visual Schema Displacement Therapy; CAPS-5, Clinician Administered PTSD Scale for DSM-5; PCL-5, PTSD Checklist for DSM-5; BDI-II, Beck Depression Inventory-II; BSI, Brief Symptom Inventory.

PTSD diagnostic status. At posttreatment, in the VSDT condition 40.0% (Z = −2.04; *p* = .021), and in the EMDR therapy condition 31.3% (Z = −1.79; *p* = .037) no longer meet the diagnostic criteria for PTSD, with no difference between these conditions (BF_01_ = 2.20). In the control condition, 7.7% lost their PTSD diagnostic status post-treatment, which was not significantly different from pretreatment (*p* >.05). At follow-up, these percentages increased further to 58.3% (VSDT), 41.2% (EMDR therapy), and 15.4% (control), although this change is only significant compared to pretreatment for VSDT (VSDT; Z = −2.27; *p* = .012) and EMDR therapy (EMDR therapy; Z = −2.27; *p* = .012), with no difference between these conditions (BF_01_ = 1.55). There is no significant change for the control condition from pretreatment to follow-up, as well as from posttreatment to follow-up for all conditions (*p*s >.05).

### Self-report measures

**Figure 3:**
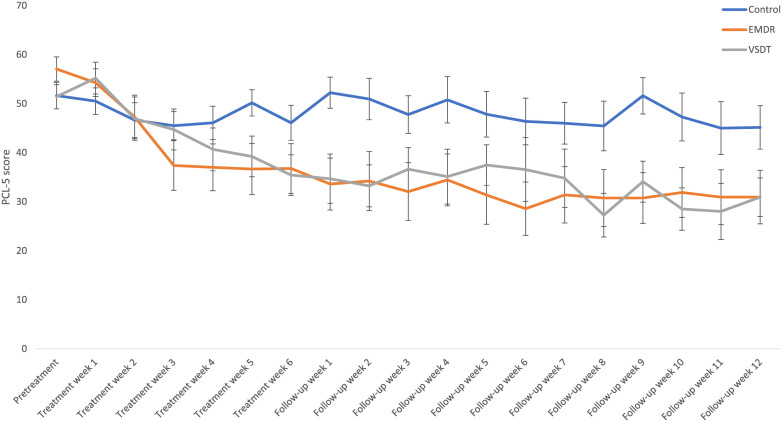
Mean (SE) PCL-5 scores specified per condition and timepoint. EMDR, Eye Movement Desensitization and Reprocessing; VSDT, Visual Schema Displacement Therapy; PCL-5, PTSD Checklist for DSM-5.

PCL-5. The Bayesian repeated measures ANOVA with condition (VSDT, EMDR therapy, Control) as independent variable and PCL-5 scores over time as the dependent variables (pretreatment, posttreatment, follow-up) shows the most support for the model including both main effects and the interaction effect (BF_M_ = 24.79) in contrast to the models including only the main effect for Time (BF_M_ = 0.36), main effect for Time and Condition (BF_M_ = 0.24), and main effect for Condition (BF_M_ = 3.91 x 10^−7^). This interaction effect is further specified by the *post hoc* ANOVA of slope differences between conditions revealing support for different decreases between conditions from pretreatment to posttreatment (BF_10_ = 21.05) and from pretreatment to follow-up (BF_10_ = 4.15). More specifically, there was a larger decrease from pretreatment to posttreatment for the EMDR therapy condition than for the control condition (BF_10_ = 22.05), as well as for the VSDT condition compared to the control condition (BF_10_ = 5.76), while differences between VSDT and EMDR therapy were not supported (BF_01_ = 2.23). For pretreatment to follow-up, similar results are found (EMDR therapy vs. Control, BF_10_ = 8.78; VSDT vs. Control, BF_10_ = 2.60; VSDT vs. EMDR therapy, BF_01_ = 2.12). No support is found for differences between the conditions from posttreatment to follow-up (BF_01_ = 2.44). For a (graphic) overview of the descriptive results, see **Table 1** and **Figure 3**.

**Figure 4:**
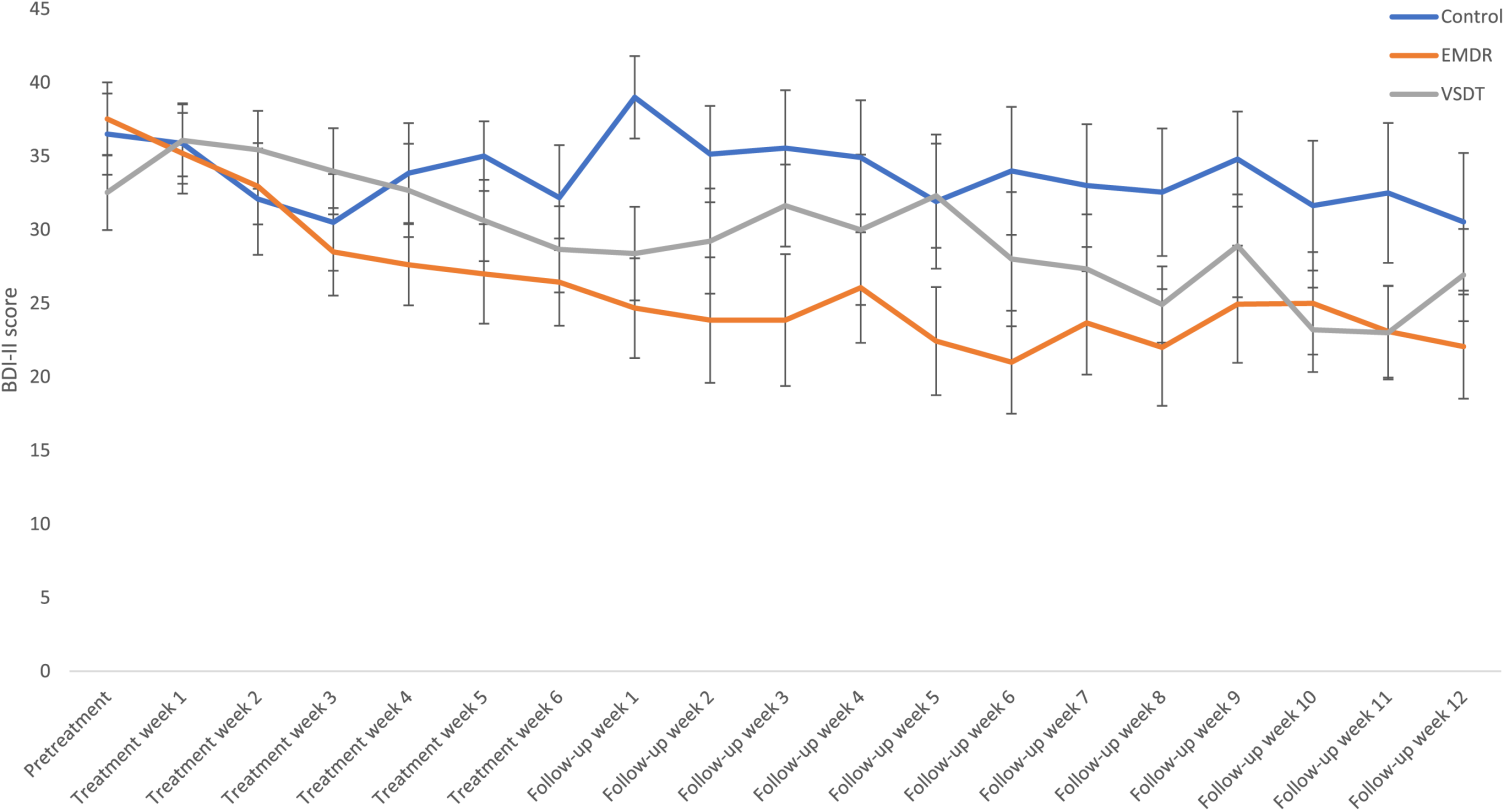
Mean (SE) BDI-II scores specified per condition and timepoint. EMDR, Eye Movement Desensitization and Reprocessing; VSDT, Visual Schema Displacement Therapy; BDI-II, Beck Depression Inventory-II.

BDI-II. The effects of treatment condition (VSDT, EMDR therapy, Control) on BDI-II scores over time (pretreatment, posttreatment, follow-up) were analyzed using a Bayesian repeated measures ANOVA, which showed most support for the model including only the main effect of Time (BF_M_ = 4.87). The model including both main effects was minorly supported as well (BF_M_ = 1.41). No support was found for the model including both main effects and interaction effect (BF_M_ = 0.93). The evidence against only a main effect for Condition was strong (BF_M_ = 1.00 x 10^−3^). The *post hoc* ANOVAs showed evidence of slope differences among the conditions from pretreatment to posttreatment (BF_10_ = 3.40), and minor support for differences between pretreatment to follow-up (BF_10_ = 1.92). Further decomposed, the analyses show a larger decrease from pretreatment to posttreatment for the EMDR therapy condition compared to the Control condition (BF_10_ = 6.68), as well as compared to the VSDT condition (BF_10_ = 1.25). No support was found for differences between VSDT and Control between these time points (BF_01_ = 2.35). Similar, although less supported, results are found between pretreatment and follow-up (EMDR therapy vs. Control, BF_10_ = 2.10; EMDR therapy vs. VSDT, BF_10_ = 2.41; VSDT vs. Control, BF_01_ = 2.70). No differences were found between conditions from posttreatment to follow-up (BF_01_ = 4.04). For a (graphic) overview of these results, see **Table 1** and **Figure 4**.

**Figure 5:**
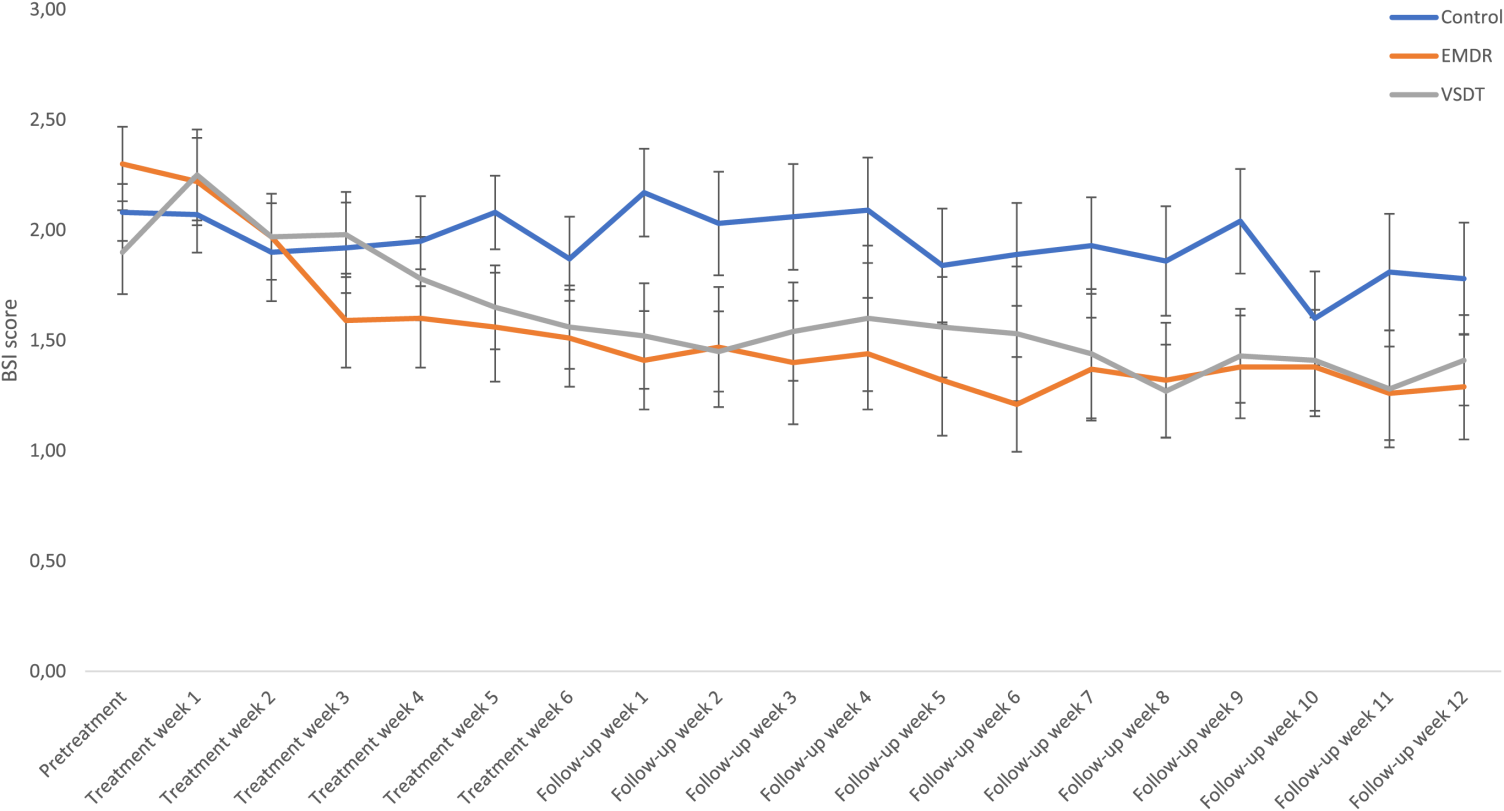
Mean (SE) BSI scores specified per condition and timepoint. EMDR, Eye Movement Desensitization and Reprocessing; VSDT, Visual Schema Displacement Therapy; BSI, Brief Symptom Inventory.

BSI. Differences between conditions (VSDT, EMDR therapy, Control) in BSI scores over time (pretreatment, posttreatment, follow-up) were analyzed using a Bayesian repeated measures ANOVA. This analysis revealed most support for the model including only the main effect of Time (BF_M_ = 3.25). The models including both main effects and the interaction effect (BF_M_ = 2.02), and only the main effects (BF_M_ = 1.10), were also supported. Major evidence against the model including only the main effect for Condition (BF_M_ = 8.06 x 10^−4^) was found. The *post hoc* analysis demonstrates support for a different decrease in BSI scores between conditions from pretreatment to posttreatment (BF_10_ = 3.07). For pretreatment to follow-up, minor support was found for differences among conditions (BF_10_ = 1.21). Broken down, the *post hoc* analyses revealed support for a larger decrease in BSI scores from pretreatment to posttreatment in the EMDR therapy condition than the control condition (BF_10_ = 6.14). Differences between VSDT vs. Control (BF_01_ = 1.30) and EMDR therapy vs. VSDT (BF_01_ = 1.53) are not supported. Similarly, from pre-treatment to follow-up, the only difference supported was the one between EMDR therapy and control (BF_10_ = 2.93; VSDT vs. Control, BF_01_ = 2.35; EMDR therapy vs. VSDT, BF_01_ = 1.28). No differences were found between conditions from posttreatment to follow-up (BF_01_ = 2.95). For a graphic overview of the descriptive results, see **Figure 5**.

### Treatment duration

A Bayesian independent multinomial test showed no difference in the number of early completers (i.e., all trauma targets treated before the maximum amount of six sessions was reached) between the EMDR therapy and the VSDT condition (BF_01_ = 2.26): 41.2% of the participants in the EMDR therapy condition needed less than six sessions compared to 33.3% in the VSDT condition. Likewise, a Bayesian ISTT with the number of sessions as the dependent variable and condition (VSDT, EMDR therapy) as independent variable showed no difference between these two groups (BF_01_ = 2.69). Participants in the EMDR therapy condition had an average of 5.1 sessions compared to 4.87 sessions in the VSDT condition.

Differences in average treatment duration per target were analyzed with a Bayesian ISTT, showing minor support for the alternative hypothesis stating that the use of VSDT led to a lower average time per target (*M* = 47.96, *SD* = 22.59) than the use of EMDR therapy (*M* = 65.48, *SD* = 24.46; BF_10_ = 1.71).

## Discussion

This study found support for a decrease in self-reported and objective PTSD symptoms for both VSDT and EMDR therapy, but no support for any differences between VSDT and EMDR therapy on subjective and objective PTSD measurements at posttreatment or follow-up, while both treatments performed better than the waitlist control condition. The reduction in PTSD symptom severity was maintained at the 3-month follow-up assessment. This finding suggests that VSDT is effective in reducing PTSD symptoms and that its effects persist over time.

One important aspect of this study was to assess the safety and feasibility of implementing VSDT as a clinical psychotherapy. To this end, no adverse events were reported during the study, indicating that VSDT can be safely conducted within a clinical context, which is consistent with previous research on VSDT in a non-clinical sample (5). Although the dropout rate of VSDT was higher than that of EMDR therapy in this study, the number of participants dropping out because of treatment-related reasons were the same between both therapies. In general, there are no high drop-out rates in the study, and in addition to the absence of any adverse events, it supports the notion that VSDT is also a feasible treatment option that can be incorporated into existing clinical practices.

In addition to determining safety, one of the key contributions of this study was its comparison of VSDT with EMDR therapy, one of the most effective evidence-based therapies for the treatment of PTSD (3). VSDT demonstrated no differences in effectiveness in comparison to EMDR therapy at the 3-month follow-up assessment, suggesting that VSDT is a potent therapy in that it is capable in producing lasting improvements in PTSD. Furthermore, the study demonstrated that a significant proportion of participants no longer met the diagnostic criteria for PTSD at posttreatment and follow-up assessments. This indicates that VSDT can not only lead to clinically meaningful improvements in PTSD symptoms, but also to remission of the disorder. Future studies should investigate the specific mechanisms underlying VSDT. Further exploration of these mechanisms may help to refine and optimize the delivery of VSDT.

In addition to reducing PTSD symptoms, both VSDT and EMDR therapy were associated with reductions in comorbid psychopathology, including depressive and general psychiatric symptoms, although EMDR seemed to be more in favor and VSDT showed no difference to either EMDR or the control condition. These findings indicate that VSDT and EMDR therapy may have broader therapeutic effects and related implications, improving overall psychological well-being in individuals with PTSD, but differences between conditions are small.

The study also explored the duration of treatment sessions and found that the average treatment time per target was lower for VSDT than for EMDR therapy. This suggests that VSDT may offer a more time-efficient treatment option. However, it is important to note that the overall number of sessions did not significantly differ between the two treatments. The question remains whether a longer treatment period will lead to more efficient outcomes for VSDT or EMDR therapy.

Despite the valuable insights gained from this study, there are limitations that should be acknowledged. First, the sample size was relatively small, and replication with a larger sample is warranted to confirm the findings. Additionally, the study primarily focused on short-term and medium-term outcomes, and long-term follow-up assessments would provide a more comprehensive understanding of the durability of treatment effects. Also, the treatment sample consisted only of females, limiting generalizability of the results, which is also true for the ongoing psychopharmacological treatment among the participants. Some strengths of the study also need to be mentioned. The treatment sessions were well-structured, with specific procedures for selecting and treating traumatic memories. Additionally, the therapists received training and supervision to ensure adherence to both study protocols, and all assessments were blinded to the treatment conditions.

In conclusion, this randomized controlled trial demonstrated that VSDT is a safe, feasible, and effective treatment for PTSD. It produced significant reductions in PTSD symptom severity, led to a substantial proportion of participants no longer meeting the diagnostic criteria for PTSD, and showed promise in addressing comorbid psychopathologies. As a time-efficient and potentially equally efficacious alternative, VSDT warrants further investigation and consideration in the treatment for PTSD.

## Data availability statement

The raw data supporting the conclusions of this article will be made available by the authors, without undue reservation.

## Ethics statement

The study was conducted according to the guidelines of the Declaration of Helsinki, the Dutch Medical Research Involving Human Subjects Act (WMO), and approved by the Medical Research Ethics Committee NedMec (Protocol ID: 19-171/D; approval date: May 15, 2019). Informed consent was obtained from all subjects involved in the study.

## Author contributions

SM: Conceptualization, Data curation, Funding acquisition, Investigation, Methodology, Resources, Software, Supervision, Validation, Writing – original draft, Writing – review & editing. TB: Data curation, Formal Analysis, Funding acquisition, Investigation, Methodology, Project administration, Software, Validation, Visualization, Writing – original draft, Writing – review & editing. AJ: Conceptualization, Investigation, Supervision, Writing – original draft, Writing – review & editing.

## Footnotes

1 For early completers of the treatment phase, an average score of the weekly measurements of the last completed treatment week and the first follow-up week was computed and imputed as value for the remaining non-completed treatment weeks to increase descriptive value. This did not influence any statistical analyses.
